# BRCA1 missense polymorphisms are associated with poor prognosis of pancreatic cancer patients in a Chinese population

**DOI:** 10.18632/oncotarget.16422

**Published:** 2017-03-21

**Authors:** Ying Zhu, Kan Zhai, Juntao Ke, Jiaoyuan Li, Yajie Gong, Yang Yang, Jianbo Tian, Yi Zhang, Danyi Zou, Xiating Peng, Jing Gong, Rong Zhong, Kun Huang, Jiang Chang, Xiaoping Miao

**Affiliations:** ^1^ Key Laboratory of Environment and Health, Ministry of Education and Ministry of Environmental Protection, Tongji Medical College, Huazhong University of Science and Technology, Wuhan, China; ^2^ Medical Research Center, Beijing Chao-Yang Hospital, Capital Medical University, Beijing, China; ^3^ Tongji School of Pharmacy, Huazhong University of Science and Technology, Wuhan, China

**Keywords:** prognosis, pancreatic cancer, BRCA, SNP

## Abstract

Pancreatic cancer is a highly lethal disease with limited prognostic marker. *BRAC1* and *BRCA2* are two classic tumor suppressor genes which play an important role in DNA repair. Somatic mutations and germline genetic variants on *BRCA1/2* have been found associated with the tumorigenesis of pancreatic cancer. However, the correlations between *BRCA1/2* polymorphism and pancreatic cancer prognosis remained unknown. In this study, we genotyped three tag missense variants on *BRCA1/2* in 603 sporadic pancreatic cancer patients in a Chinese population. We found rs1799966 on *BRCA1* was associated with poor prognosis of pancreatic cancer patients with hazard ratio being 1.23 (95% CI: 1.09–1.40, *P* = 0.0010). Further stratification analyses showed that significant correlation was particularly in locally advanced stage patients with hazard ratio being 1.36 (95% CI: 1.13–1.64, *P* = 0.0014), but not in patients in local stage (*P* = 0.1139) or metastatic stage (*P* = 0.5185). Two missense variants (rs766173 and rs144848) on *BRAC2* showed no significant correlation with pancreatic cancer patients’ overall survival. In conclusion, we identified a germline missense variant on *BRAC1* significantly associated with poor prognosis of pancreatic cancer patients with locally advanced stage. These results may contribute to the precision medicine of this disease.

## INTRODUCTION

Pancreatic cancer is the thirteenth most common cancer in the world with mortality closely parallels incidence [1, 2]. Despite decades of efforts, there are still few early detection methods and effective treatments of this disease. Nevertheless, patient survival times are varied and only partly explained by traditional clinical and pathological features [3]. Accumulating evidences have shown that germline variation such as single nucleotide polymorphisms (SNP) can not only affect cancer susceptibility [4, 5], but also confer patients with different prognosis in multiple cancers [6–8]. Genetic variation relating to transcription regulation and DNA damage repair have effects on patient survival particularly.

*BRCA1* and *BRCA2* are two recognized tumor suppressor genes, for the encoded proteins play important roles in transcription and DNA repair of double-stranded breaks via homologous recombination [9, 10]. Mutations in *BRCA1/2* have been confirmed to give rise to multiple cancers including pancreatic adenocarcinoma [11–14]. Moreover, prognostic value of germline *BRCA* mutations have also been found in breast, ovarian, prostate and other cancers [15–19]. Germline variants on *BRCA2* have also been identified to be associated with pancreatic cancer risk [20]. However, correlation between variants on *BRCA* and pancreatic cancer patients’ survival are rarely studied.

In the present study, we investigated the impact of germline missense variants on *BRCA1*/*2* and overall survival (OS) of pancreatic cancer patients. Three tag missense polymorphisms (rs1799966 on *BRCA1*; rs766173 and rs144848 on *BRCA2*) were genotyped in 603 pancreatic cancer patients in a Chinese population.

## RESULTS

### Characteristics of study subjects

The characteristics of the 603 pancreatic cancer patients are shown in Table 1. Median follow-up time for these patients was 15 months. 523 (86.7%) patients died during our follow-up time with median survival time (MST) being 7.4 months. As expected, disease stage was strongly associated with patients’ overall survival (*P* < 0.0001) with MST being 13.7, 8.1 and 4.4 months for patients with local, locally advanced and metastatic stage, respectively. Patients’ gender, age, smoking or drinking status showed no significant correlation with patients’ overall survival.

**Table 1:** Selected characteristics and overall survival of study subjects

	No. (%)	MST	*P*^†^
Vital status			
Dead	523 (86.7)		
Alive	80 (13.3)		
Gender			0.6639
Male	369 (61.2)	7.3	
Female	234 (38.8)	7.4	
Age^‡^			0.0848
≤ 61	309 (51.2)	7.7	
> 61	294 (48.8)	7.1	
Smoking status			0.1920
Yes	140 (23.2)	7.0	
No	463 (76.8)	7.5	
Drinking status			0.7561
Yes	108 (17.9)	7.6	
No	495 (82.1)	7.3	
Clinical stage			< 0.0001
Local	137 (22.7)	13.7	
Locally advanced	268 (44.5)	8.1	
Metastatic	198 (32.8)	4.4	

Note: MST, median survival time (months).

†*P* values were calculated using the log-rank test.

‡Median age was 61 years among all cases.

### Genetic variants on *BRCA1* is associated with pancreatic patients’ overall survival

**Figure 1:**
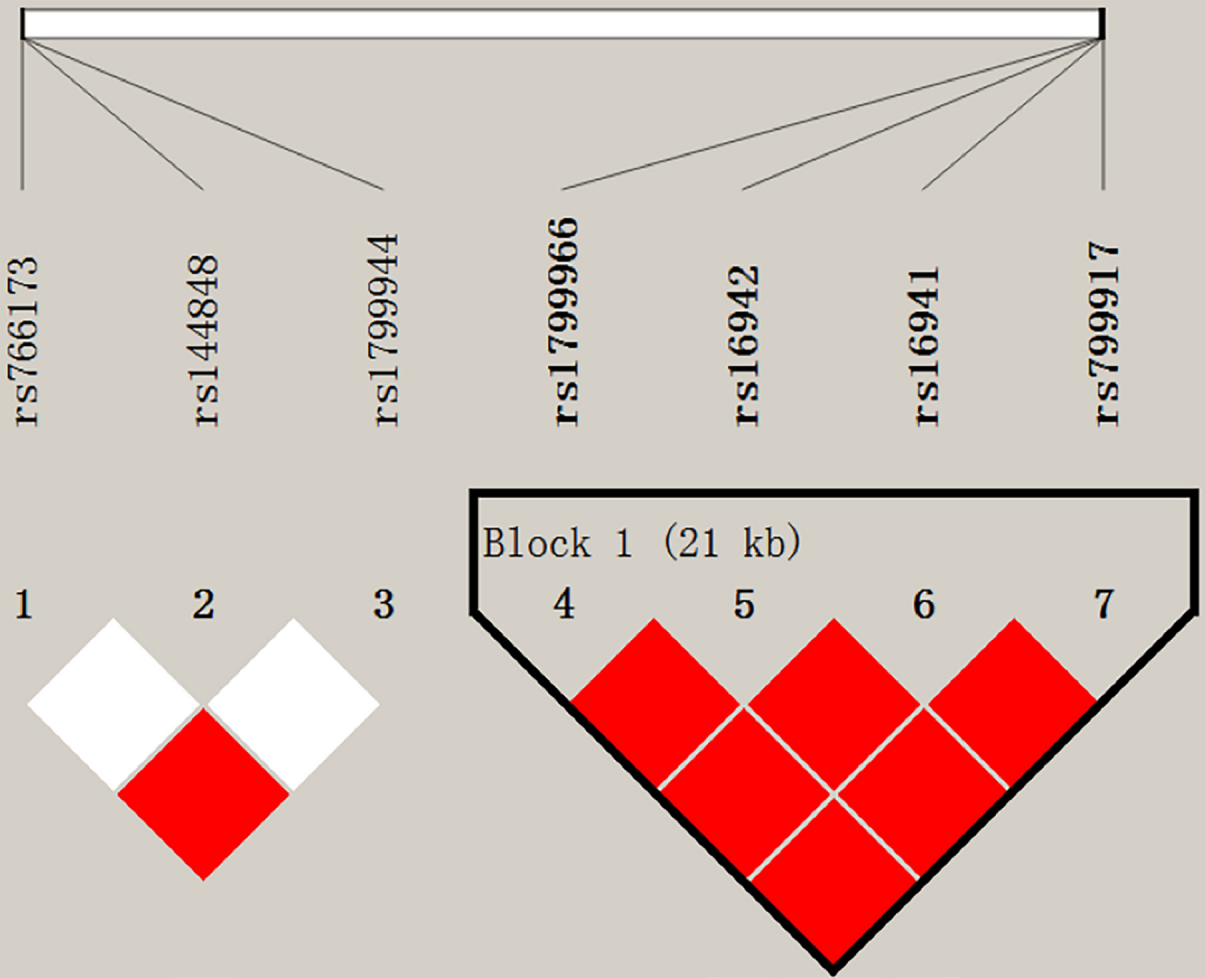
Linkage disequilibrium (LD) of SNPs at the *BRCA1/2* gene locus rs766173 and rs1799944 on *BRCA2* are in complete linkage disequilibrium (LD), but independent with the rest SNP rs144848. Four SNPs (rs1799966, rs16942, rs16941 and rs799917) on *BRCA1* are in complete LD. The LD plot was based on 1000 genome phase 3 data among the Han Chinese in Beijing (CHB). White diamond indicates hardly no evidence of LD (*r*^2^ = 0.028), while red indicates strong evidence (*r*^2^ = 1).

**Figure 2:**
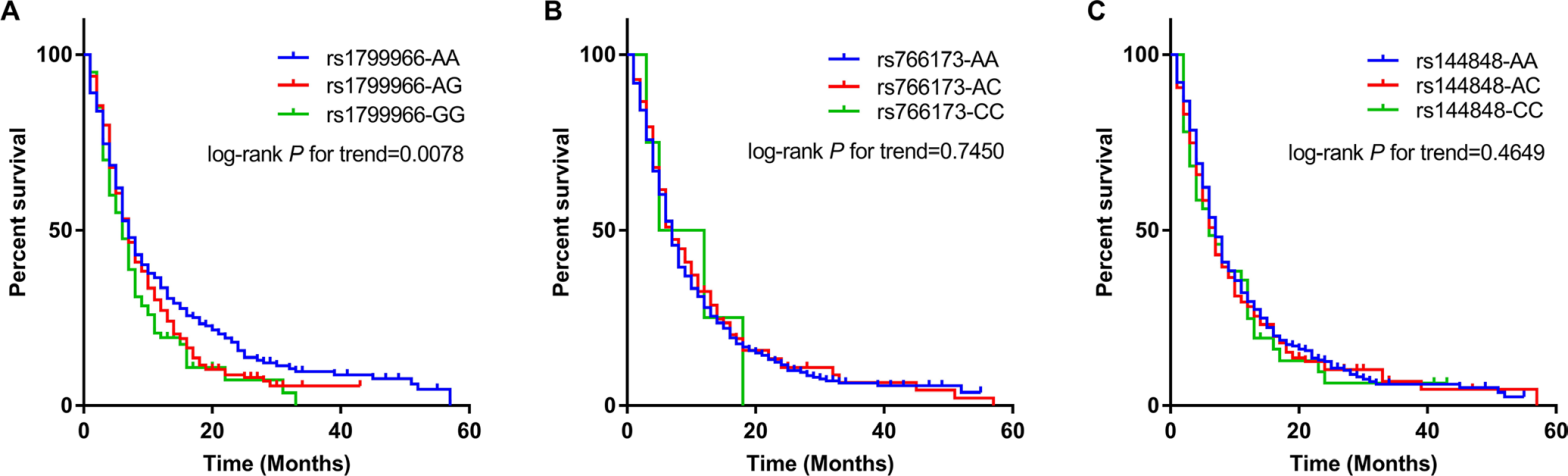
Kaplan–Meier curves of overall survival by rs1799966 (**A**), rs766173 (**B**) and rs144848 (**C**) genotypes. *P* values were calculated using Log-rank test for trend in an additive model.

We retrieved a total of 24 missense variants with global MAF greater than 0.01 on *BRCA1* and *BRCA2* (Supplementary Table 1). Among these variants, seven (four on *BRCA1* and three on *BRCA2*) are common variants with MAF > 0.05 in CHB (Chinese Han, Beijing) population (Supplementary Table 1). All the four missense variants on *BRCA1* (rs1799966, rs16942, rs16941, rs799917) are in perfect linkage disequilibrium (LD) (Figure 1). For *BRCA2*, rs766173 and rs1799944 are in complete LD, but independent with the rest variant rs144848 (Figure 1). Therefore, considering the LD relations and function prediction clues provided by SIFT and Polyphen, we selected rs1799966 (*BRCA1*), rs766173 and rs144848 (*BRCA2*) as tag SNPs for genotying and further survival analysis. As a result, we observed that rs1799966 (*BRCA1*) was significantly associated with pancreatic cancer patients’ overall survival with HR being 1.24 under an additive model (95% CI: 1.09–1.40, *P* = 0.0010) (Table 2 and Figure 2). Comparing with patients with AA genotype, the patients with AG genotype and GG genotype were significantly associated with poor prognosis with median survival time (MST) being 7.5 month (HR = 1.32, 95% CI: 1.09–1.59, *P* = 0.0044) and 6.7 month (HR = 1.41, 95% CI: 1.07–1.85, *P* = 0.0143), respectively (Table 2). However, two variants on *BRCA2* (rs766173 and rs144848) showed no significant correlation with prognosis of pancreatic cancer patients with *P* values under an additive model being 0.8579 and 0.1798, respectively (Table 2 and Figure 2).

**Table 2:** Association between variants on BRCA1/2 and PDAC prognosis

SNP, Gene	No. (%)	MST	HR (95% CI)	*P*^†^
**rs1799966, BRCA1**				
**AA**	**248 (41.1)**	**7.6**	**1.00 (Reference)**	
**AG**	**275 (45.6)**	**7.5**	**1.32 (1.09–1.59)**	**0.0044**
**GG**	**80 (13.3)**	**6.7**	**1.41 (1.07–1.85)**	**0.0143**
**Additive model**			**1.23 (1.09–1.40)**	**0.0010**
rs766173, *BRCA2*				
AA	487 (80.6)	7.4	1.00 (Reference)	
AC	112 (18.6)	7.3	1.01 (0.81–1.26)	0.9302
CC	4 (0.7)	12.0	1.15 (0.43–3.10)	0.7767
Additive model			1.02 (0.83–1.25)	0.8579
rs144848, *BRCA2*				
AA	327 (54.2)	7.7	1.00 (Reference)	
AC	235 (39.0)	7.1	1.08 (0.90–1.30)	0.3924
CC	41 (6.8)	6.8	1.23 (0.86–1.76)	0.2644
Additive model			1.10 (0.96–1.27)	0.1798

Note: SNP, single nucleotide polymorphism; MST, median survival time (months); HR, hazard ratio; CI: confidence interval.

†*P* values were calculated using Cox regression adjusting for gender, age, smoking, drinking status and clinical stage.

### Stratification analyses of rs1799966 with pancreatic cancer patients’ overall survival

**Figure 3:**
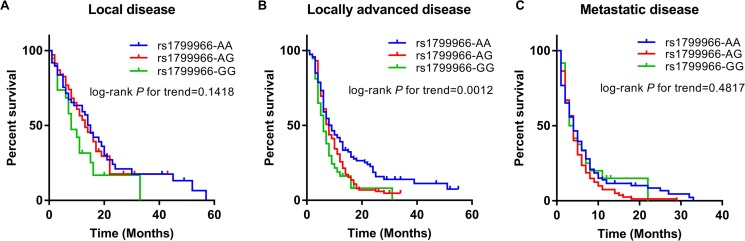
Kaplan–Meier curves by rs1799966 genotypes in different disease stages *P* values were calculated using Log-rank test for trend in an additive model in patients with local stage (**A**), locally advanced stage (**B**) and metastatic stage (**C**), respectively.

We further performed stratified analyses by gender age, smoking status, drinking status and clinical stage to evaluate the effects of rs1799966 (c.4837A>G [p.Ser1613Gly]) on pancreatic cancer patients’ overall survival (Table 3 and Supplementary Table 2). The rs1799966 showed significant association with overall survival of pancreatic cancer patients with different age group (*P* = 0.0095 and *P* = 0.0370 for patients ≤ 61 years and > 61 years, respectively), smoking status (*P* = 0.0025 and *P* = 0.0210 for smoker and non-smoker, respectively) and drinking status (*P* = 0.0144 and *P* = 0.0117 for drinker and non-drinker, respectively). The association was also significant in males (*P* = 0.0014) but not in females (*P* = 0.1654). Among patients with different clinical stage, the rs1799966 was particularly significant associated with poor prognosis of locally advanced stage patients with an HR being 1.36 under an additive model (95% CI: 1.13–1.64, *P* = 0.0014) (Table 3 and Figure 3) and no significant correlation between rs1799966 and patients’ overall survival was showed in patients with local (*P* = 0.1139) or metastatic stage (*P* = 0.5185) (Table 3 and Figure 3).

**Table 3:** Stratification analyses of rs1799966 with PDAC patients’ prognosis in different stages

	No. (%)	MST	HR (95% CI)^†^	*P*^†^
Local disease (*N* = 137)				
AA	49 (35.8)	15.0	1.00 (Reference)	
AG	69 (50.3)	13.7	1.07 (0.67–1.69)	0.7858
GG	19 (13.9)	8.8	1.70 (0.93–3.13)	0.0869
Additive model			1.28 (0.94–1.74)	0.1139
**Locally advanced disease (*N* = 268)**				
**AA**	**113 (42.2)**	**9.0**	**1.00 (Reference)**	
**AG**	**118 (44.0)**	**8.1**	**1.45 (1.09–1.94)**	**0.0104**
**GG**	**37 (13.8)**	**6.6**	**1.66 (1.11–2.50)**	**0.0147**
**Additive model**			**1.36 (1.13–1.64)**	**0.0014**
Metastatic disease (*N* = 198)				
AA	86 (43.4)	4.6	1.00 (Reference)	
AG	88 (44.5)	4.4	1.25 (1.92–1.71)	0.1534
GG	24 (12.1)	4.0	0.99 (0.61–1.61)	0.9703
Additive model			1.07 (0.87–1.32)	0.5185

Note: MST, median survival time (months); HR, hazard ratio; CI: confidence interval.

†*P* values were calculated using Cox regression adjusting for gender, age, smoking and drinking status.

## DISCUSSION

In this study, we interrogate the correlation between germline missense variants on *BRCA1*/*2* and pancreatic cancer patients’ overall survival. Through genotyping of three tag missense variants on *BRCA1/2* in 603 Chinese pancreatic cancer patients, we discovered the prognostic value of rs1799966 (c.4837A>G [p.Ser1613Gly]) on *BRCA1*. Further stratification analyses showed that the association was particularly in patients with local advanced stage, but not patients in local or metastatic stage.

*BRCA1* and *BRCA2* are two classic tumor suppressor genes that play important roles not only in tumorigenesis but also cancer progression and outcome. A meta-analysis among 297,402 patients revealed that *BRCA* mutations were associated with worse overall survival in breast cancer patients [21]. Similar findings were also reported in prostate cancer that significant longer cause-specific overall survival has been reported for *BRCA* mutation non-carriers compared with carriers [22]. However, in the context of pancreatic cancer, there still lacks convincing epidemiology data or association study for the prognostic effects of *BRCA* germline mutation. In this present study, we found a tag missense variant (c.4837A>G [p.Ser1613Gly]) on *BRCA1* significantly associated with poor prognosis of pancreatic cancer patients in a Chinese population. This variant can lead to amino acid change from serine to glycine (p.Ser1613Gly), and was predicted to have a possibly damaging of BRCA1 protein function by SIFT or Polyphen. The *BRCA1* has many important functions in maintaining the normal physiology of cell and organisms [23, 24]. Its major role is involved in the regulation of cell cycle checkpoints in response to DNA damage, including the repair of DNA double-strand breaks (DSB) via homologous recombination [10, 25]. *BRCA1* deficient cells lacking HR activity could accumulate DNA DSB, resulting in genomic instability, malignant transformation and progression [26]. Accordingly, we conjectured that the missense variants rs1799966 may hinder the performance of *BRCA1* in the above biological process thus conferring pancreatic cancer patients in different genotypes with different risk of deterioration and survival time.

In the stratification analyses, we only observed that rs1799966 was significantly associated with survival of locally advanced stage patients. For local stage disease, patients carrying risk allele and genotype also had poorer survival (AG, HR = 1.07; GG, HR = 1.70). However, associations were not significant might because relatively small sample size, and other treatments such as chemotherapy and radiotherapy after surgery which might influence survival times. As for distant metastatic disease, the prognosis are extremely poor for both risk allele carriers and non-carriers, that the genetic effects on patients’ survival might not be obvious.

To the best of our knowledge, it was the first association study of *BRCA* germline variants and pancreatic cancer patients’ overall survival. Our work highlighted the important prognostic and predictive value of *BRCA1* germline variants, which had been mostly regarded as a risk biomarker of pancreatic cancer though. There were still some limitations for this study. First, our findings need to be validate in replication cohorts with larger sample sizes or other populations in the future. Second, the underlying biological mechanism between rs1799966 and pancreatic cancer patients’ survival needs further investigation.

In conclusion, we identified a missense variant rs1799966 on *BRCA1* associated with a worse overall survival of pancreatic cancer patients, especially in patients with locally advanced stage. Our findings not only lead to new insights into the progression of pancreatic cancer, but also suggest a target for therapy and potential biomarker for prognosis prediction.

## MATERIALS AND METHODS

### Study subjects

Pancreatic cancer patients were recruited from Tongji Hospital of Huazhong University of Science and Technology, Wuhan, China. A subset of individuals were included in our previous studies [27, 28]. The diagnosis of pancreatic cancer was confirmed by two local pathologists according to the World Health Organization classification [29]. Demographic, clinical and survival information were obtained from the medical records and telephone interview. Informed consent was obtained from all participants at recruitment, and this study was approved by the ethics committee of Tongji Medical College, Huazhong University of Science and Technology.

### SNP selection

We utilized Ensembl genome browser (http://asia.ensembl.org/index.html) to retrieve all the missense variants on *BRCA1* and *BRCA2*, with global minor allele frequency (MAF) greater than 0.01 (using 1000 genome phase 3 data). Among which, we focused on the common variants in CHB (Han Chinese in Beijing, China) population with MAF in CHB greater than 0.05 (a total of seven variants met the standard, four on *BRCA1* and three on *BRCA2*. Supplementary Table 1.). Then we took advantage of SIFT and PolyPhen to predict variant effects on protein function, and meanwhile measured linkage disequilibrium (LD) relations between each pair of variants using 1000 genome phase 3 data (Figure 1). Synthesizing the above results, we selected 3 tagSNPs (rs1799966 on *BRCA1*; rs766173 and rs144848 on *BRCA2*) with most likely functional as candidate for genotyping.

### Genotyping

At recruitment, 2 mL peripheral venous blood sample was collected from each subject with informed consent. Genomic DNA was extracted using the RelaxGene Blood DNA System DP319-02 (TIANGEN). The above three candidate SNPs were subsequently genotyped using a TaqMan assay on the ABI PRISM 7900 HT platform (Applied Biosystems, Inc.). The sequences of primers and TaqMan probes for genotyping candidate SNPs are listed in Supplementary Table 3. For quality control, 5% duplicate samples were independently reanalyzed in a blinded fashion. The call rate of each SNP was over 95%.

### Statistical analysis

The overall survival time was defined as the time from pancreatic cancer diagnosis to either death or the last known date alive. Cox proportional hazards regression under a log-additive genetic model with adjustment for gender, age, smoking and drinking status and stage of disease was used to measure the effects of candidate SNPs on survival. Haploview software was used to determine pair-wise LD relations. Kaplan–Meier survival estimates were plotted and *P* values were assessed using the log-rank test for trend. Survival analyses were performed with R (3.3.0) using “coxph” function in “Survival package”. For all analyses, statistical significance was set at *P* < 0.05 and all tests were two sided.

## SUPPLEMENTARY TABLES


