# Supplementary material for: BRCA1 missense polymorphisms are associated with poor prognosis of pancreatic cancer patients in a Chinese population
Source: Oncotarget. 2017 Mar 21;8(22):36033–9. doi: 10.18632/oncotarget.16422 (PMC5482636; doi:10.18632/oncotarget.16422)
Supplement: Supplementary file 1 [file oncotarget-08-36033-s001.pdf]

BRCA1 missense polymorphisms are associated with poor prognosis of pancreatic cancer patients in a Chinese population

Supplementary Materials

Supplementary Table 1: Missense variants on *BRCA1/2* with global MAF > 0.01

SNP	Gene	Chr	Position	Alleles	Global MAF	CHB MAF	AA	SIFT_class	Polyphen_class
rs1799967	*BRCA1*	17	43070958	G/A	0.011	0.000	M/I	tolerated	benign
**rs1799966**	***BRCA1***	**17**	**43071077**	**A/G**	**0.356**	**0.350**	**S/G**	**deleterious**	**possibly damaging**
rs16942	*BRCA1*	17	43091983	A/G	0.353	0.350	K/R	tolerated	benign
rs2227945	*BRCA1*	17	43092113	A/G	0.010	0.000	S/G	tolerated	benign
rs4986852	*BRCA1*	17	43092412	G/A	0.010	0.000	S/N	tolerated	benign
rs16941	*BRCA1*	17	43092418	A/G	0.336	0.350	E/G	tolerated	benign
rs799917	*BRCA1*	17	43092919	C/T	0.456	0.350	P/L	tolerated	benign
rs56082113	*BRCA1*	17	43093073	A/G	0.010	0.000	K/E	tolerated	benign
rs4986850	*BRCA1*	17	43093454	G/A	0.034	0.000	D/N	deleterious	benign
rs1799950	*BRCA1*	17	43094464	A/G	0.022	0.000	Q/R	deleterious	probably damaging
rs1800062	*BRCA1*	17	43115746	G/T	0.013	0.039	K/N	deleterious	probably damaging
**rs766173**	***BRCA2***	**13**	**32332343**	**A/C**	**0.074**	**0.136**	**N/H**	**deleterious**	**benign**
**rs144848**	***BRCA2***	**13**	**32332592**	**A/C**	**0.249**	**0.252**	**N/H**	**tolerated**	**benign**
rs1799944	*BRCA2*	13	32337326	A/G	0.080	0.136	N/D	tolerated	benign
rs4986860	*BRCA2*	13	32355172	A/G	0.010	0.000	H/R	tolerated	benign
rs169547	*BRCA2*	13	32355250	C/T	0.024	0.000	A/V	tolerated	benign
rs11571707	*BRCA2*	13	32356461	T/C	0.016	0.000	I/T	tolerated	benign
rs11571769	*BRCA2*	13	32379413	G/A	0.010	0.000	A/T	deleterious	probably damaging
rs1801426	*BRCA2*	13	32398747	A/G	0.045	0.044	I/V	tolerated	benign

Note: SNP, single nucleotide polymorphism; Chr, chromosome; MAF, minor allele frequency; CHB, Chinese Han Beijing; SIFT and Polyphen are algorithms

Supplementary Table 2: Stratification analyses of rs1799966 and PDAC patients' prognosis with different gender, age, smoking and drinking status

	No. (%)	MST	HR (95% CI)†	*P*
Male (*N* = 369)				
AA	146 (39.6)	7.3	1.00 (Reference)	
AG	177 (48.0)	7.4	1.65 (1.28–2.12)	0.0001
GG	46 (12.4)	6.8	1.39 (0.96–2.01)	0.0815
Additive model			1.30 (1.11–1.53)	0.0014
Female (*N* = 234)				
AA	102 (43.6)	7.7	1.00 (Reference)	
AG	98 (41.9)	7.8	0.99 (0.73–1.33)	0.9424
GG	34 (14.5)	5.5	1.45 (0.95–2.22)	0.0812
Additive model			1.16 (0.94–1.42)	0.1654
≤ 61 years (*N* = 309)				
AA	124 (40.1)	8.3	1.00 (Reference)	
AG	146 (47.3)	7.8	1.33 (1.01–1.73)	0.0405
GG	39 (12.6)	6.3	1.52 (1.02–2.27)	0.0394
Additive model			1.27 (1.06–1.53)	0.0095
> 61 years (*N* = 294)				
AA	124 (42.2)	7.3	1.00 (Reference)	
AG	129 (43.9)	6.9	1.30 (0.99–1.70)	0.0603
GG	41 (13.9)	6.9	1.40 (0.95–2.06)	0.0857
Additive model			1.21 (1.01–1.44)	0.0370
Smoker (*N* = 140)				
AA	60 (42.9)	7.8	1.00 (Reference)	
AG	58 (41.4)	7.0	1.71 (1.12–2.61)	0.0131
GG	22 (15.7)	6.5	1.84 (1.05–3.21)	0.0324
Additive model			1.49 (1.15–1.93)	0.0025
Non-smoker (*N* = 463)				
AA	188 (40.6)	7.5	1.00 (Reference)	
AG	217 (46.9)	7.7	1.24 (1.00–1.54)	0.0484
GG	58 (12.5)	7.0	1.34 (0.97–1.85)	0.0768
Additive model			1.19 (1.03–1.38)	0.0210
Drinker (*N* = 108)				
AA	45 (41.7)	8.5	1.00 (Reference)	
AG	46 (42.6)	7.3	2.01 (1.20–3.37)	0.0079
GG	17(15.7)	6.8	1.75 (0.91–3.38)	0.0948
Additive model			1.46 (1.08–1.97)	0.0144
Non-drinker (*N* = 495)				
AA	203 (41.0)	7.4	1.00 (Reference)	
AG	229 (46.3)	7.5	1.26 (1.02–1.55)	0.0317
GG	63 (12.7)	6.5	1.36 (1.00–1.85)	0.0519
Additive model			1.20 (1.04–1.38)	0.0117

Note: MST, median survival time (months); HR, hazard ratio; CI: confidence interval.

Supplementary Table 3: The sequences of primers and TaqMan probes for genotyped SNPs

SNP	Name	Sequence (5′-3′)
rs1799966	Forward Primer	TGGCAACATACCATCTTCAA
rs1799966	Reverse Primer	CATTATACCCAGCAGTATCAGTAG
rs1799966	Forward Probe	FAM-ATCTGCCCAGAGTCCA-MGB
rs1799966	Reverse Probe	HEX-ATCTGCCCAGGGTCCA-MGB
rs766173	Forward Primer	AACTATCTTCTTCAGAGGTA
rs766173	Reverse Primer	AGACCACATTGGAAAGT
rs766173	Forward Probe	FAM-TCTTCTAGGACATTTGGCA-MGB
rs766173	Reverse Probe	FAM-TCTTCTAGGACATGTGGCA-MGB
rs144848	Forward Primer	GGCCAAAGACGGTACAAC
rs144848	Reverse Primer	CCATGAAGCAAACGCTGAT
rs144848	Forward Probe	FAM-CTGATTTGCTACATTTGA-MGB
rs144848	Reverse Probe	FAM-CTGATGTGCTACATTTGA-MGB
